# Chronic Subdural Hematoma Masquerading as Intracranial Hypotension

**DOI:** 10.7759/cureus.89431

**Published:** 2025-08-05

**Authors:** Brandon Sharkey, Vito Lucarelli, Ethan Kosco, Maya Mendonsa, Austin Lawrence, Kevin Reinard

**Affiliations:** 1 Department of Neurological Surgery, University of Toledo College of Medicine and Life Sciences, Toledo, USA; 2 Department of Neurological Surgery, ProMedica Toledo Hospital, Toledo, USA

**Keywords:** arterial embolization, intracranial hypotension, middle meningeal artery (mma), non-traumatic, subdural hematoma

## Abstract

Spontaneous intracranial hypotension (SIH) is caused by cerebrospinal fluid leak and has an incidence of approximately five per 100,000 person-years. SIH leads to a range of clinical symptoms, from debilitating postural headaches to subdural hematomas (SDHs). The pathophysiology of the disease is not fully understood, but has garnered interest in recent years. Although CT myelogram and pan-spine MRIs are currently considered the gold standard for diagnosis of SIH, the variable sensitivity (48-76%) and wide range of hematoma size (2-30 mm) warrant further investigation into improved diagnostic and treatment options. We present the case of a patient with non-traumatic bilateral SDHs and clinical findings suggestive of intracranial hypotension and CSF leak. The patient initially presented with typical signs and symptoms of SIH and resulting SDH. However, her clinical course was highly unusual given the sudden resolution of her symptoms despite receiving interventions that were not believed to have repaired the underlying pathology. This unique presentation of an SDH encourages the need for larger prospective studies of patients with SIH and SDH and the exploration of the role and efficacy of middle meningeal artery (MMA) embolization. This procedure is a new and promising treatment for SDH regardless of the etiology.

## Introduction

Spontaneous intracranial hypotension (SIH) is an uncommon yet established cause of sudden-onset postural headaches in previously asymptomatic patients. While postural headaches are considered a hallmark clinical symptom of SIH, their intensity and duration are widely variable and sometimes absent altogether. The clinical presentation of the disease generally encompasses a wide range of symptomatology [1]. The underlying pathophysiology of SIH has historically been poorly understood, but it is agreed upon that, ultimately, a cerebrospinal fluid (CSF) leak is the causal agent [2,3,4,5]. SIH can be treated conservatively or with an epidural blood patch (EBP) for persistent cases. While EBP has a reasonably moderate success rate with single or serial administration, surgical repair may be necessary in persistent and severe cases where EBP is unsuccessful, assuming the leak is localized. The location of the CSF leak is frequently unable to be determined despite the use of multiple imaging modalities, further complicating treatment [6,7,8].

Subdural hematoma (SDH) is a common complication of SIH due to decreased intracranial pressure leading to brain sagging, followed by tearing of the bridging veins and subsequent hematoma formation [9]. Patients with SIH who develop unilateral or bilateral SDHs are subjected to further neurologic complications, which can result in permanent debilitation. If the underlying CSF leak cannot be repaired promptly, surgical evacuation of the SDH may be necessary [10]. It is noted, however, that without fixing the underlying leak, a recurrent hematoma and persistent neurologic symptoms are likely [11]. The risks of recurrent hematoma formation have been proven to be mitigated with embolization of the middle meningeal artery (MMA) in conjunction with evacuation of the hematoma. This is a minimally invasive procedure that involves blocking the blood supply to the main contributor to the hematoma and encouraging its resorption. MMA embolization has recently gained acknowledgment for its efficacy in treating chronic SDH, its low incidence of post-operative complications, and preventing further recurrence. Together, this significantly reduces the need for high-risk and invasive surgical intervention. However, there is still debate regarding the optimal timing and the best treatment for large SDHs [12,13].

Here, we report a case in which a patient with suspected SIH and bilateral SDH presented to our hospital with persistent postural headaches status post 2 EBPs. Following burr hole evacuation of the hematoma and MMA embolization, her neurologic symptoms rapidly resolved with no recurrence or complication despite failure to address any underlying CSF leak.

## Case presentation

**Figure 1:**
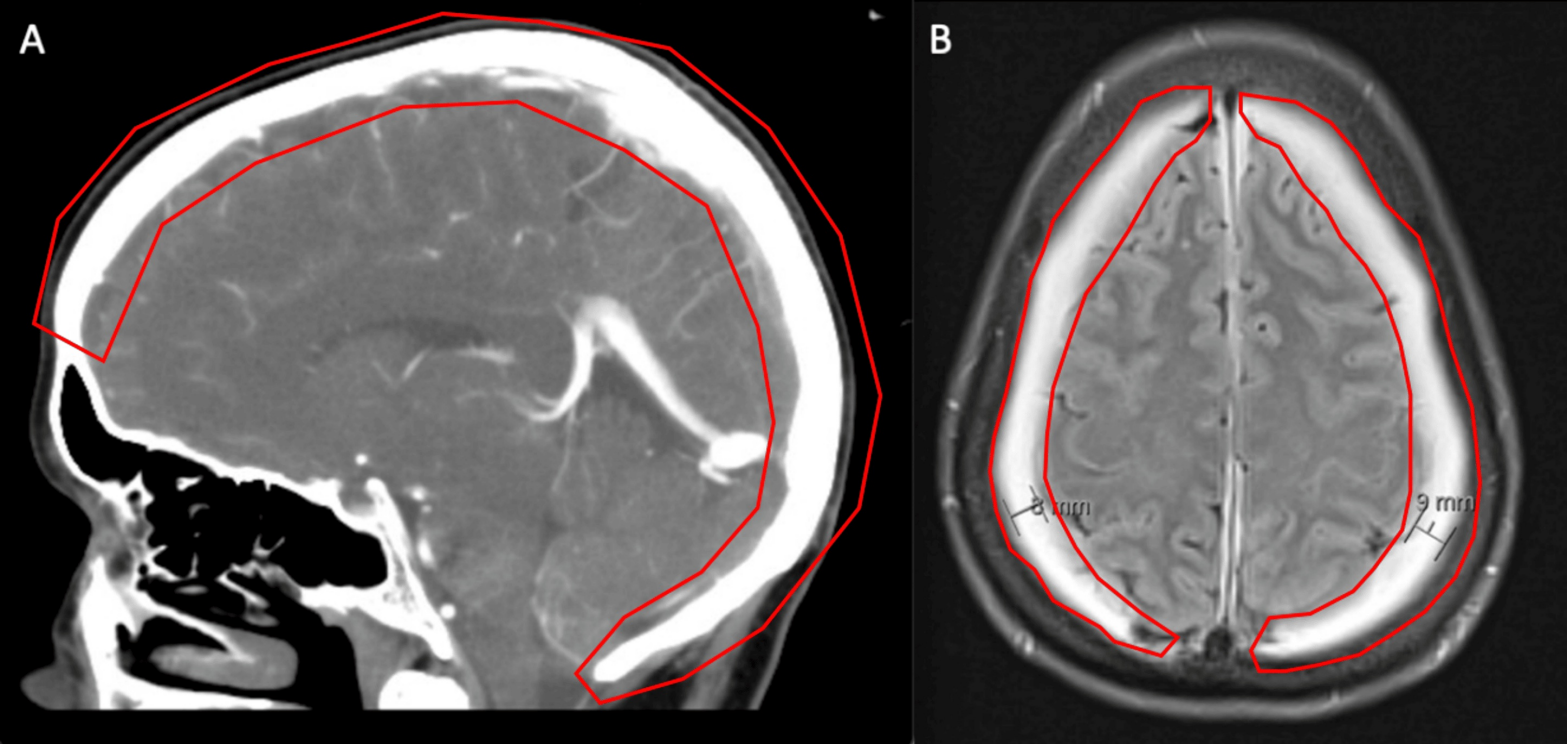
Sagittal CT and MRI scan emphasizing subdural hemorrhage. A) Sagittal contrast-enhanced CT view highlighting holo-hemispheric collections (red). B) Axial T2-weighted MRI showcasing bilateral holo-hemispheric collections and bilateral subdural hemorrhage (red). The sizes of the collections were 8 mm on the right and 9 mm on the left.

A 56-year-old female initially presented to the emergency department for two weeks of nonspecific headaches and high at-home blood pressure measurements with associated nausea, vomiting, dizziness, and no inciting trauma. A day before presenting to the ER, she began taking lisinopril and Norvasc as per her primary care physician for hypertension. Initial computed tomographic (CT) angiogram and magnetic resonance imaging (MRI) revealed small ventricles and a prominent pituitary, both findings indicative of intracranial hypotension as the underlying etiology. Additional findings appreciated on imaging were bilateral holohemispheric collections and bilateral subdural hemorrhage with hyperacute signal (Fig. 1).

Magnetic resonance venography (MRV) was performed and ruled out the presence of a venous sinus thrombosis. Based on the findings and the patient’s clinical presentation, plans were made for the patient to receive an epidural blood patch following a CT myelogram of the cervical, thoracic, and lumbar spine to visualize a spontaneous CSF leak of the spine. The patient was subsequently started on a migraine cocktail (magnesium sulfate, Reglan, Benadryl), received a caffeine infusion, and had her bed placed in the Trendelenburg position.

**Figure 2:**
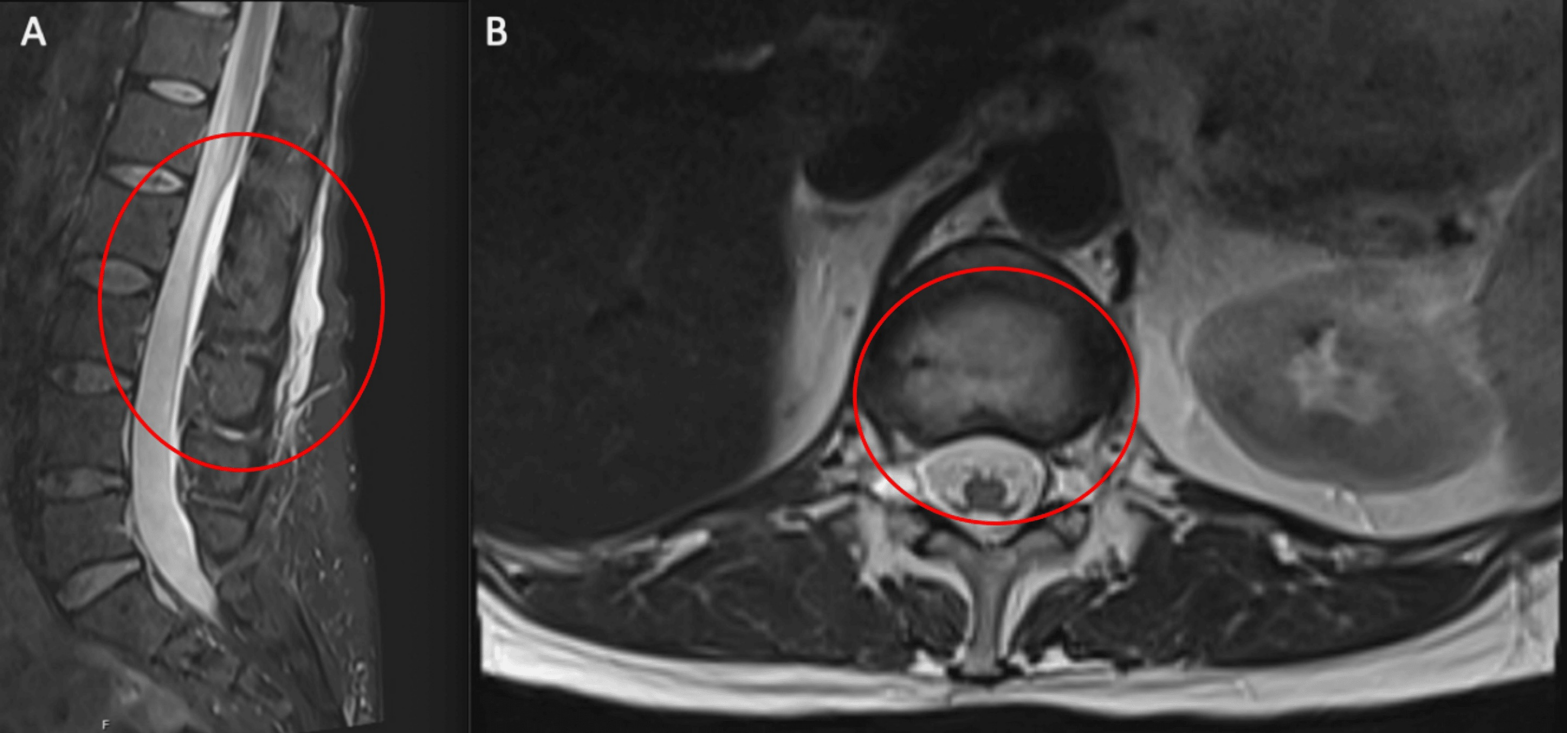
Sagittal and axial T2 MRI pan spine and follow-up imaging. A) Sagittal T2-weighted MRI pan spine demonstrating a large perineural cyst at the right T12-L1 level (red). B) Follow-up axial T2-weighted MRI two days later demonstrating the large perineural cyst at the right T12-L1 level (red).

Due to concerns of worsening symptoms of a CSF leak with a CT myelogram, an MRI pan spine was performed instead to visualize a leak. Follow-up imaging two days later demonstrated a moderate to large perineural cyst at the right T12-L1 level and additional smaller perineural cysts within the lower thoracic and lumbar spine that could potentially serve as the etiology for a spontaneous CSF leak (Fig. 2).

The next day, the patient received an epidural blood patch and had a complete resolution of her headache a few hours later. She was then discharged the following day with plans for a one-month follow-up and repeat MRI studies.

The patient was seen in the office by a neurologist nine days following her discharge to have a short-term disability, and Family Medical Leave Act (FMLA) forms were completed. At this point, her headaches had not returned, but she endorsed hypersomnia, fatigue, and complained of brain fog. She was given education on Tarlov cysts and encouraged to avoid Valsalva maneuvers, heavy lifting, and contact sports. Two weeks later, the patient returned to the office with new complaints of positional headaches that are worse while lying down and improved while standing, developing incontinence of urine, changes to her gait, and short-term memory loss. Her symptoms continued to worsen and prevented her from returning to work.

**Figure 3:**
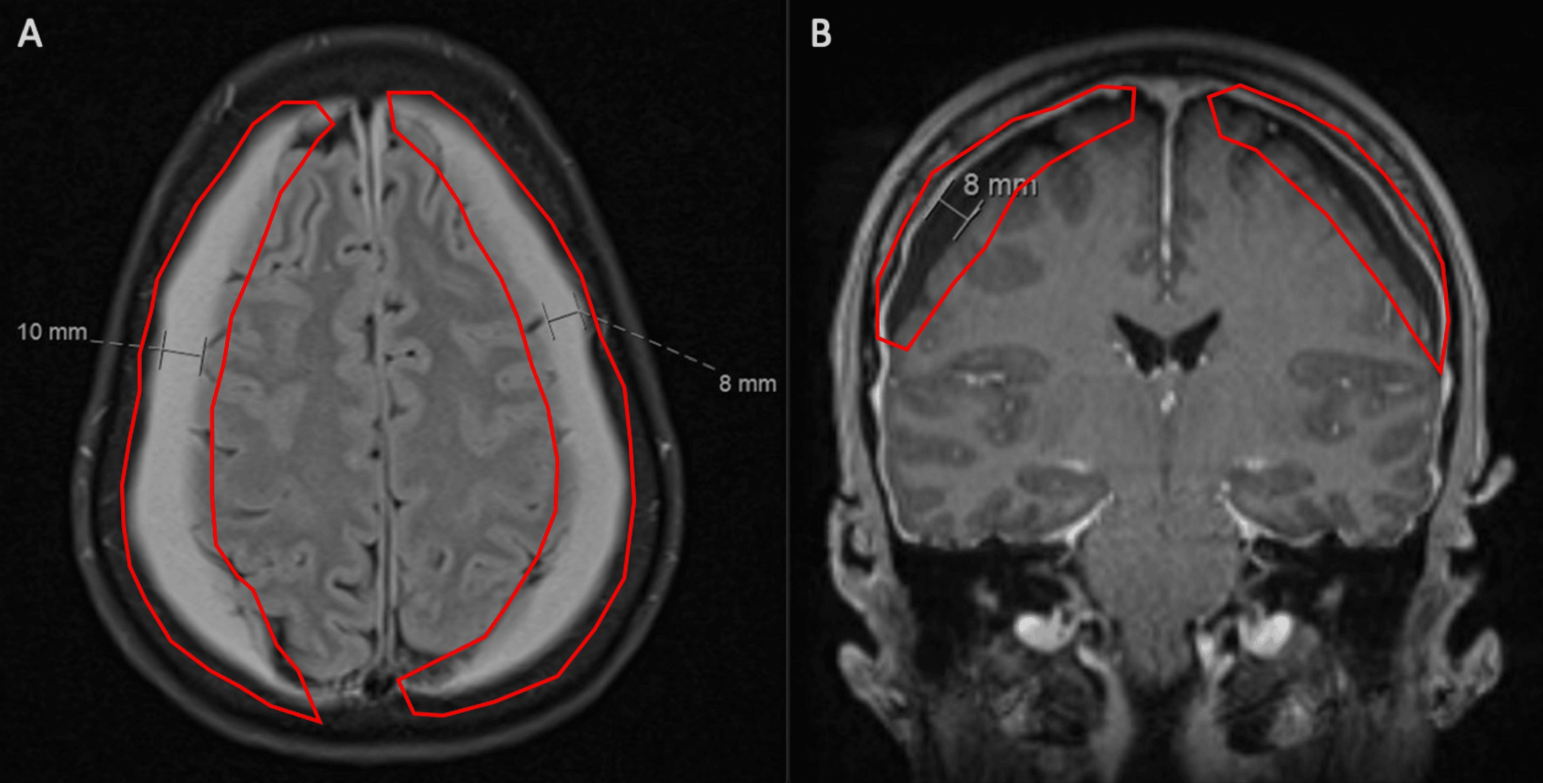
Axial and coronal follow-up MRI scans. A) Axial follow-up T1-weighted MRI demonstrating stigmata of intracranial hypotension, including minimal worsening of pachymeningeal enhancement and thickening (red). The sizes of the collections increased to 10 mm on the right and 8 mm on the left. B) Coronal follow-up T1-weighted MRI demonstrating stigmata of intracranial hypotension, including minimal worsening of pachymeningeal enhancement and thickening (red).

A trial of modafinil was started to provide short-term symptom relief while labs were drawn. The results revealed a severe vitamin B12 deficiency (nadir of 151). The patient was subsequently started on sublingual vitamin B12 and began receiving B12 injections. A second opinion was sought at an outside institution, where the patient was informed that her symptoms could take three to six months to resolve. The institution had concerns that the patient may have an underlying connective tissue disorder due to the presence of perineural cysts and an uncle with a history of Marfan syndrome. A repeat epidural blood patch was recommended if either follow-up imaging demonstrated no improvement of her bilateral subdural hemorrhages or her headaches worsened. Follow-up imaging five weeks after her initial imaging demonstrated stigmata of intracranial hypotension with minimal worsening of pachymeningeal enhancement and thickening and the size of the overlying bilateral cerebral convexity subdural hemorrhages (Fig. 3).

The patient was seen in the office by neurosurgery one week later, where plans were made to have a second epidural blood patch and a CT myelogram of the spine to visualize the site of the leak. She presented to the hospital later that day and received a second epidural blood patch.

**Figure 4:**
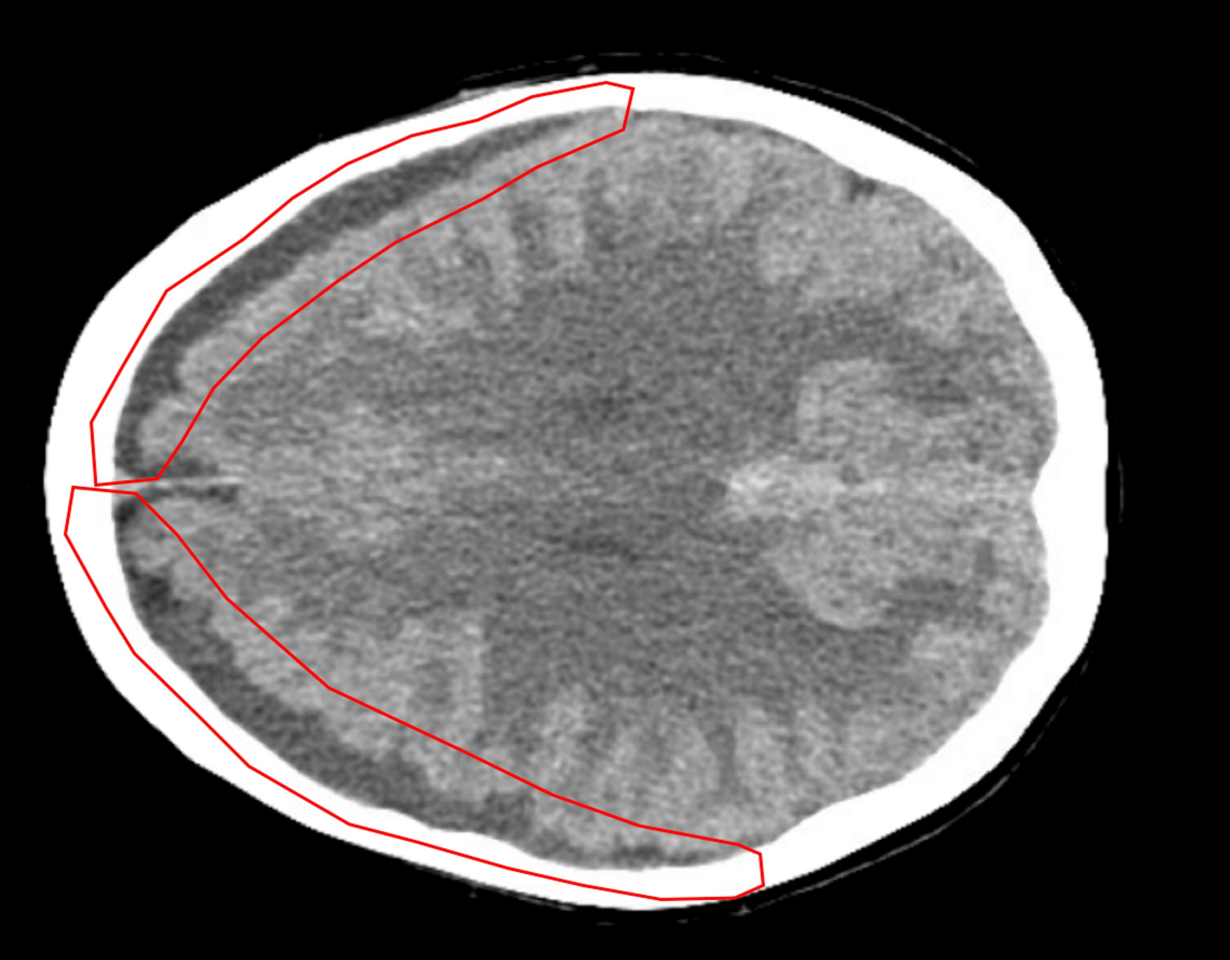
Axial CT Imagining demonstrating asymmetric density of the subdural fluid collections (red). This suggests subacute blood products on the right without evidence of acute hemorrhage.

Following her second epidural blood patch, she was headache-free for approximately 12 days before she began reporting a return of head pain that improved while lying down, a change from her previous headaches, which prompted a CT study of the brain. Computed tomographic imaging was performed 24 days following her second epidural blood patch and two days following the return of her symptoms. Imagining demonstrated asymmetric density of the subdural fluid collections, suggesting subacute blood products on the right without evidence of acute hemorrhage and continued findings of intracranial hypotension. It also demonstrated that the size of the extra-axial fluid had not changed from the previous MRI studies (Fig. 4).

She was later seen in the office by neurology, and a trial of Ambien was started as the hope was of improved quality of sleep; she had experienced fewer memory complaints. She was also previously advised to remain on bed rest with the head of her bed slightly elevated as much as possible during a 24-hour period, which she reported improved her headaches.

**Figure 5:**
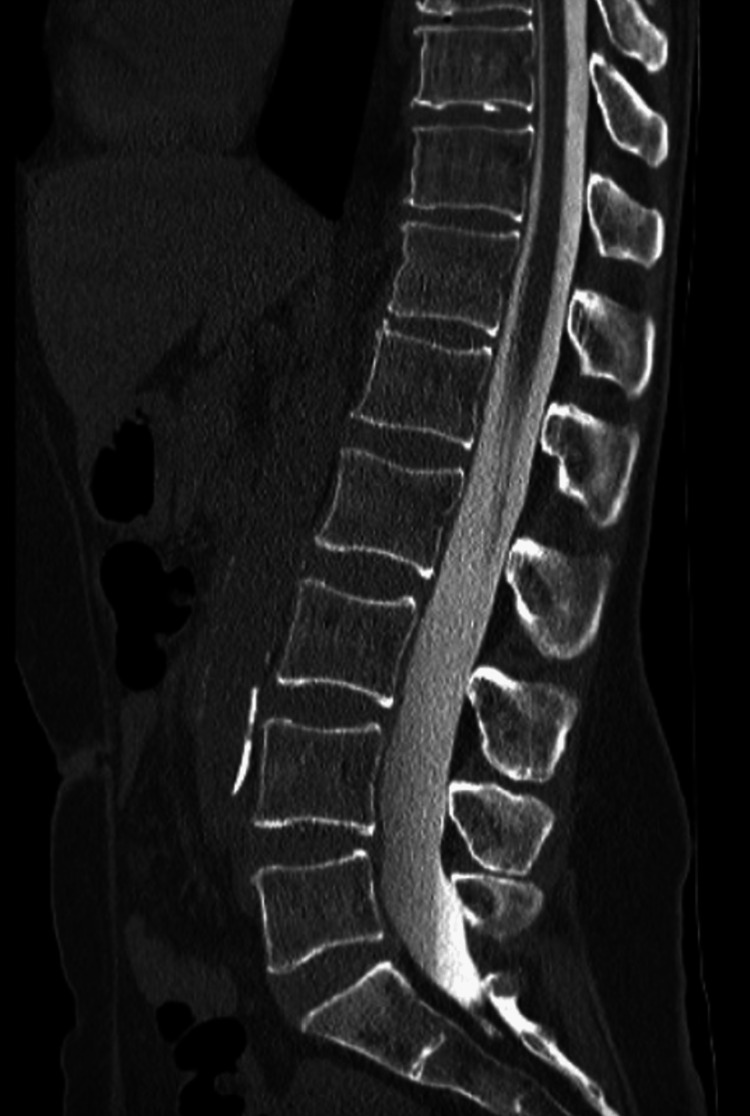
CT myelogram of the lumbar spine performed 20 days following her previous brain CT demonstrates excellent opacification of the thecal sac with contrast and no evidence of a CSF leak or subdural collections.

A CT myelogram of the lumbar spine was performed 20 days following her previous brain CT that demonstrated excellent opacification of the thecal sac with contrast and no evidence of a CSF leak or subdural collections (Fig. 5).

A few days later, she was seen in the office by neurosurgery, where her husband explained that she had been mainly on bed rest over the past four weeks. He also reported that her physical and cognitive symptoms had returned and worsened.

**Figure 6:**
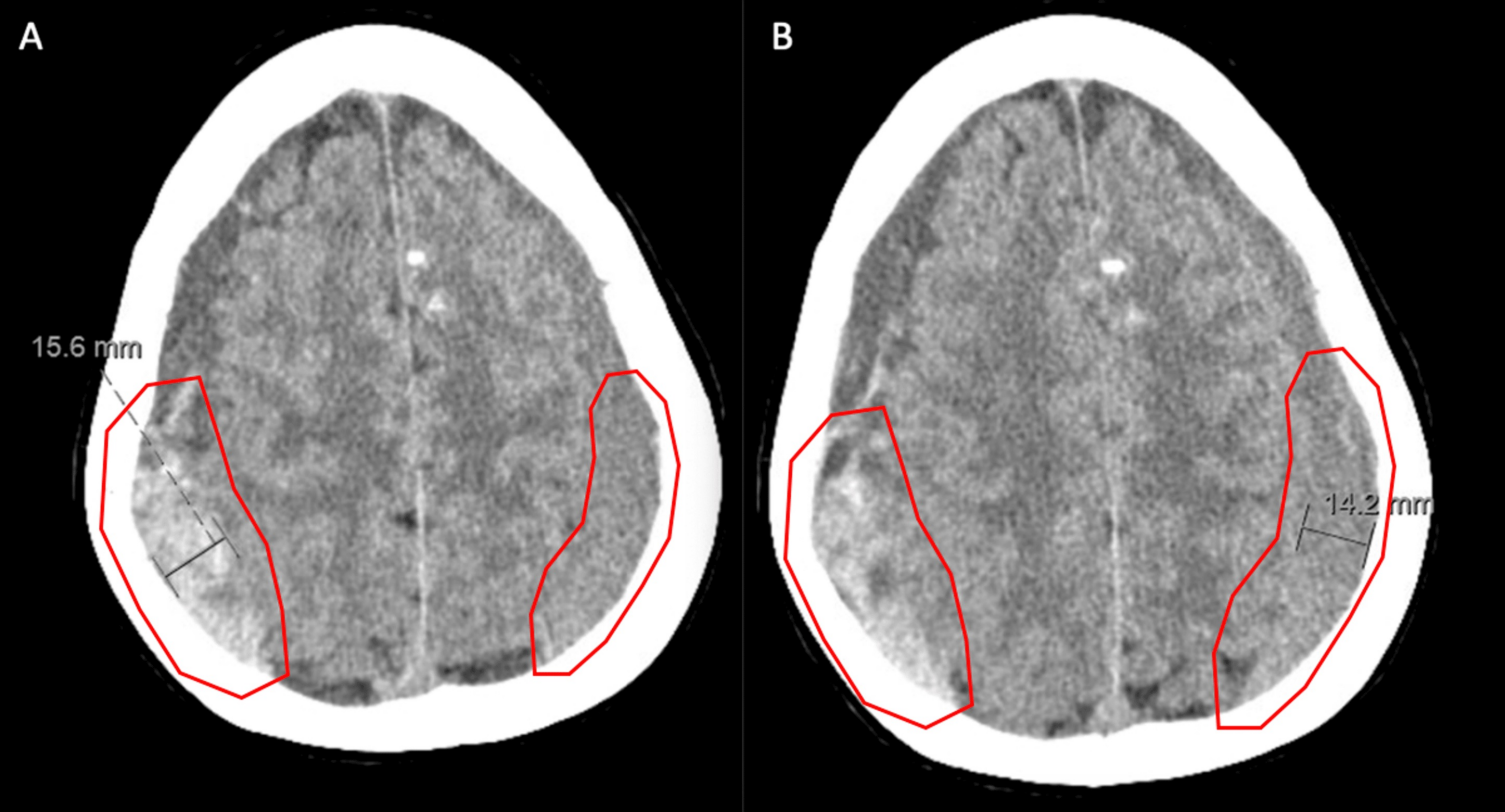
Axial brian CTs performed the same day. A) and B) Axial brian CTs performed the same day demonstrated progression of previously noted bilateral subdural hematomas (red). The sizes of the collections further increased to 16.6 mm on the right and 14.2 mm on the left.

Recommendations were then made for her to be admitted to the hospital for repeat imaging and a potential third epidural blood patch. A CT myelogram of the cervical and thoracic spine was performed four days later and demonstrated no definitive evidence of a CSF leak or extradural contrast collection. A repeat brain CT done the same day demonstrated the progression of previously noted bilateral subdural hematomas (Fig. 6).

**Figure 7:**
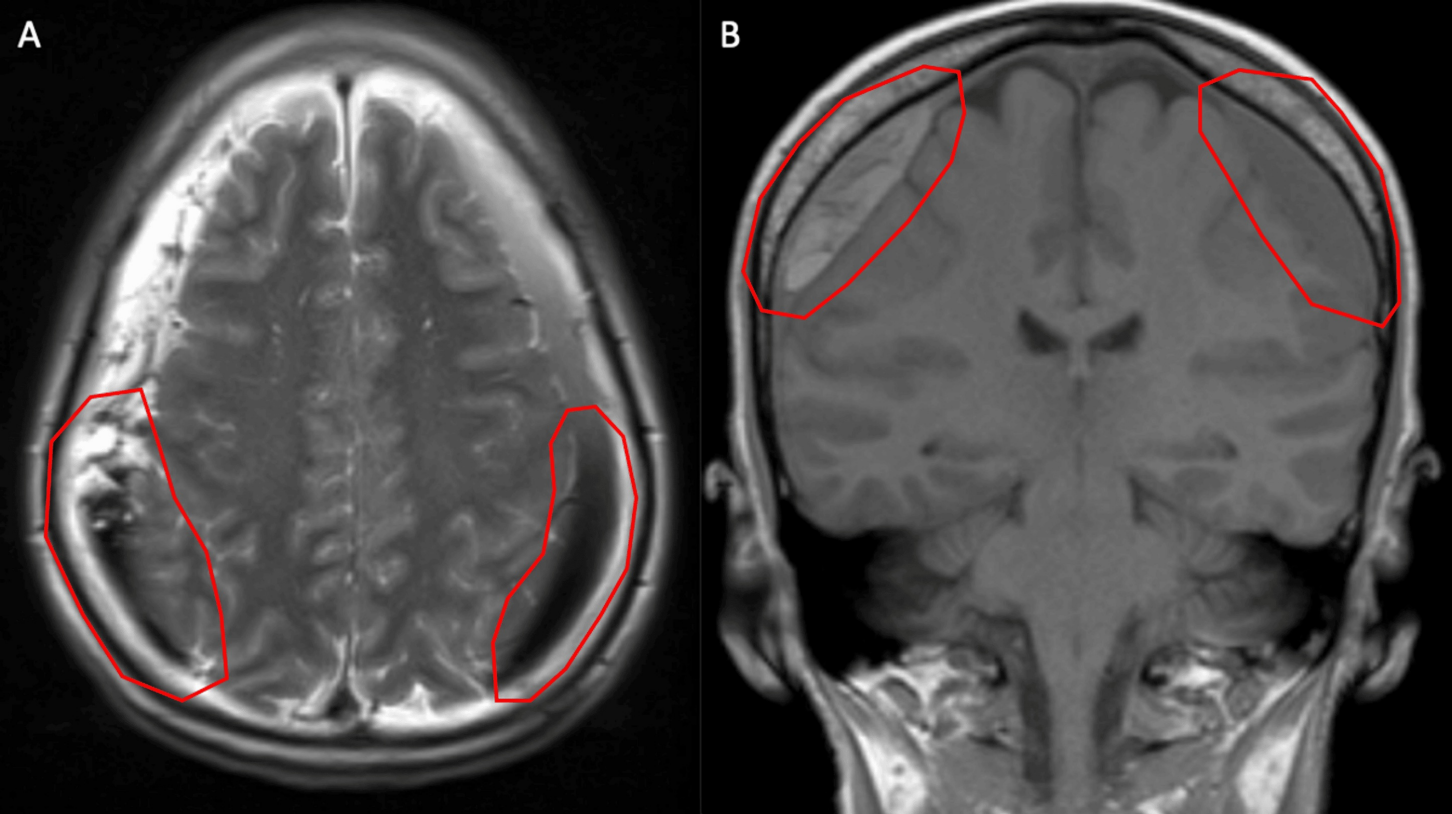
Axial and coronal MRIs of subdural hematomas A) Axial MRI demonstrating bilateral subdural hematomas with mild localized mass effect upon the cerebral hemispheres (red). B) Coronal MRI demonstrating bilateral subdural hematomas with mild localized mass effect upon the cerebral hemispheres (red).

MR imaging of the brain the next day demonstrated bilateral subdural hematomas as above with mild localized mass effect upon the cerebral hemispheres (Fig. 7).

The decision was made to take the patient to the operating room two days later for bilateral subdural hematoma burr hole evacuation due to the failure of two previous blood patches, a negative full spinal myelogram, and serial imaging of the brain demonstrating chronic subdural fluid collections with formation of pseudomembranes and brain compression. Bilateral subdural drains were placed and tunneled during the procedure. As soon as postoperative day 1, the patient began appreciating significant improvement and relief from her headaches. Three days following her surgery, the patient underwent bilateral middle meningeal artery embolization. The patient was discharged the next day as she was alert and oriented with clear and appropriate speech, a steady gait, and had complete resolution of her headaches and other symptoms that had first begun three months prior. The post-op course has remained uneventful with significant improvement of her preoperative symptoms involving cognitive decline, and the patient has returned to work and is performing daily activities.

## Discussion

It is not unusual to diagnose intracranial hypotension following a lumbar puncture or neurosurgery [14]. SIH, on the other hand, remains a poorly understood phenomenon with a wide variety of clinical presentations [1]. In one study, the data demonstrated that for most patients with spontaneous intracranial hypotension, the diagnosis is missed initially, as many other pathologies can mimic intracranial hypotension [15]. A spontaneous spinal CSF leak remains the prominent cause of SHI and can result from either dural rents or arachnoid cysts [2,3,4,5]. An important and frequent sequela of SIH is the development of SDHs that result from brain sagging with meningeal traction and subsequent tearing of bridging veins [9]. The patient in this case presented similarly to most patients with SIH: positional headaches and eventual formation of SDHs. A recent review of the literature identified orthostatic headaches as the most common manifestation of SIH [16]. In the initial evaluation of suspected SIH, an MRI of the brain is performed to look for findings such as diffuse dural or pachymeningeal enhancement, which our patient’s imaging demonstrated [6].

When approaching the management of spontaneous intracranial hypotension, many available treatment options vary based on an identified underlying cause that may have led to the SIH. As outlined earlier, CSF leaks are a prominent cause of SHI. In this case, a CT myelogram and MRI pan spine were performed to determine whether or not there was a leak and its location. Historically, CT myelography has been regarded as the preferred method for identifying and determining the location of a CSF leak [15]. No leak could be identified on either CT myelogram or MRI pan spine, which is not unusual as the presence of extradural CSF could only be detected in 48-76% of patients in a recent meta-analysis [17]. Following the implementation of conservative measures such as bed rest with upright positioning, oral hydration, and caffeine intake, the mainstay of SIH treatment is an epidural blood patch. The success rate of non-targeted epidural blood patching varies greatly, with reports ranging from 30% to 70% for the first patch [18,19,20]. It is widely recognized and reported that most patients with SIH may require multiple epidural blood patches for adequate resolution of symptoms. Although the patient described in our report achieved symptomatic relief following both epidural blood patches, the relief was short-lived and had worsening symptoms soon after. Due to the nature of the patient’s response to both conservative and EBP treatment, evacuation of their bilateral CSDHs was then performed. In the setting of an unidentified CSF leak, there is a significant concern for recurrence or worsening subdural hemorrhage following an initial surgical evacuation [9]. Although the patient's symptoms were partially explained by correction of her vitamin B12 level, her rapid resolution of symptoms following surgical intervention and improved postoperative imaging support the success of the procedure.

This case highlights the rare presentation of a chronic subdural hematoma masquerading as intracranial hypotension. Several case reports have also supported the diagnostic and treatment pathway described in this manuscript and describe the complications associated with the conditions, such as subsequent herniation and Duret hemorrhage [21]. Unlike these previous studies, our patient had sustained resolution without CSF leak repair. The embolization procedure may have disrupted the inflammatory cascade driving SDH progression. The overlap of symptoms between these conditions can make diagnosis challenging and highlights the need for further research to better understand the relationship between CSDHs, CSF leaks, and intracranial hypotension. It also emphasizes the importance of considering alternative diagnoses when patients present with symptoms commonly associated with intracranial hypotension.

## Conclusions

This case illustrates the diagnostic and therapeutic challenges of SIH, particularly when complicated by bilateral chronic subdural hematomas (CSDHs) and an unlocalized CSF leak. The wide variability in symptom presentation and often inconclusive imaging findings make SIH a difficult condition to recognize early, frequently delaying appropriate treatment. The case also underscores the limitations of conservative and non-targeted treatments when the CSF leak cannot be identified and demonstrates the value of early surgical intervention in select patients. Despite initial improvement with two epidural blood patches, our patient experienced recurrent symptoms and worsening hematomas, ultimately requiring surgical evacuation followed by MMA embolization. This combined approach led to full and sustained resolution of symptoms. Ultimately, MMA embolization has emerged as a minimally invasive option to reduce recurrence by targeting the vascular supply of the dura. The procedure should be considered in refractory SDH cases, even without CSF leak localization. This case challenges the dogma that CSF leak repair is mandatory for SDH resolution. Further randomized clinical trials examining the effectiveness of MMA embolization in cases of SIH-related CSDH are needed to better define treatment strategies for patients who do not respond to standard therapies.
